# Detection of genetic variations in the *GDF9* and *BMP15* genes in Kazakh meat–wool sheep

**DOI:** 10.5194/aab-66-401-2023

**Published:** 2023-12-07

**Authors:** Makpal Amandykova, Zarina Orazymbetova, Tilek Kapassuly, Altynay Kozhakhmet, Saltanat Khamzina, Kairat Iskakov, Kairat Dossybayev

**Affiliations:** 1 Laboratory of Animal Genetics and Cytogenetics, Institute of Genetics and Physiology SC MSHE RK, Almaty, 050060, Kazakhstan; 2 Faculty of Biology and Biotechnology, Al-Farabi Kazakh National University, Almaty, 050040, Kazakhstan; 3 Kazakh Research Institute of Livestock and Fodder Production, Almaty 050035, Kazakhstan

## Abstract

Kazakh meat–wool sheep are of great interest because of the intrabreed multifetal type's high productivity of 140 %–160 %. Genes encoding growth differentiation factor-9 (*GDF9*) and bone morphogenetic protein 15 (*BMP15*) are promising candidates for studying sheep productivity, as they affect fertility in mammals, including sheep. Thus, the purpose of this study was to assess the fertility of the Kazakh meat–wool sheep breed based on *GDF9* and *BMP15* candidate genes of fecundity for the selection of animals with valuable genotypes. We selected 300 heads of the Kazakh meat–wool sheep breed from two populations for PCR-RFLP (polymerase chain reaction–restriction fragment length polymorphism) analysis, 15 of which were subsequently used for sequencing of exon regions of the *GDF9* and *BMP15* genes. The sheep populations were tested for G1 and G8 mutations of the *GDF9* gene and B2 and B4 mutations of the *BMP15* gene. The PCR-RFLP analysis revealed that 59 (19.7 %) of the 300 Kazakh meat–wool breed sheep were heterozygous carriers of the G1 mutation (genotype AG) of the *GDF9* gene, and sequencing analysis supported these results. The comparative phylogenetic analysis showed a clear separation of Kazakh meat–wool sheep wild types and carriers of the G1 mutation. This mutation was reported to have a relationship with the animals' litter size in other sheep breeds. For this reason, similar relationships should be investigated in Kazakh meat–wool sheep. However, G8, B2, and B4 mutations were not detected among the studied animal populations, showing that these mutations are not characteristic of the Kazakh meat–wool sheep breed.

## 1 Introduction

Sheep breeding is one of the leading livestock sectors in the Republic of Kazakhstan. In 2011, after long-term selection and breeding work, scientists and practitioners, sheep breeders in Kazakhstan, tested a multiparous intrabreed type of the Kazakh meat–wool sheep breed. This type was created by crossing Kazakh fine-wool ewes with Finnish Landrace rams. The fertility of ewes is 140 %–160 % (Makhatov et al., 2021).

Sheep fertility is a principal breeding trait, as it largely determines the economic effect of animal husbandry. High precocity, combined with high fertility, can provide a quick return on investment in the industry. Today, four major fecundity genes affecting follicle growth and ovulation frequency are known. Three loci belonging to the TGF- β (transforming growth factor β ) family include the Booroola *BMPR1B* gene (bone morphogenetic protein receptor 1B), *BMP15* (bone morphogenetic protein 15), and *GDF9* (growth differentiation factor 9) (Monsivais et al., 2017), and the fourth is the *B4GALNT2* gene encoding a glycosylation enzyme that does not belong to the BMP (bone morphogenetic protein) family (Ben et al., 2019). These genes regulate the expression and secretion of hormones that affect follicle growth and ovulation frequency (McNatty et al., 2003). The *GDF9* and *BMP15* genes belong to the TGF- β family, a transforming growth factor, and are principal factors in folliculogenesis regulation and ovulation in sheep (Liu et al., 2019; Chu et al., 2005; Dong et al., 1996; Nilsson and Skinner, 2002). The polymorphism of these genes may significantly impact the productivity of sheep, including their reproductive function and livestock output.

*GDF9* gene mutations (*FecG* ${}^{H}$ , *FecG* ${}^{T}$ , *FecG* ${}^{E}$ , *FecG* ${}^{F}$ , and *FecG* ${}^{V})$ led to hyperproliferation in heterozygous sheep and sterility in homozygous sheep (Melo et al., 2008; El Fiky et al., 2017; Mullen et al., 2013). Mutations in the *FecG* ${}^{E}$ and *FecG* ${}^{F}$ of the *GDF9* gene affect fecundity traits like ovulation rate and litter size, while mutations in *FecG* ${}^{H}$ , *FecG* ${}^{T}$ , and *FecG* ${}^{V}$ cause increased ovulation rate and litter size in heterozygote ewes and infertility in homozygote carriers (Muhaghegh Dolatabady and Habibizad, 2019). Many studies of the *GDF9* gene mutation aim at revealing its association with an increase in litter size. For example, studies in short-tailed sheep have shown that individuals with simultaneous mutations in the *GDF9* and *BMP15* genes have a higher ovulation frequency than animals carrying only one mutation each (Hanrahan et al., 2004).

*BMP15* was the first gene to be associated with fertility. All *BMP15* mutations (*FecX* ${}^{I}$ , *FecX* ${}^{H}$ , *FecX* ${}^{B}$ , and *FecX* ${}^{G})$ result in the same phenotype. Heterozygous sheep are highly fertile, while homozygous females are infertile because of inhibition of follicle development at the primary stage (Hanrahan et al., 2004; Galloway et al., 2000; Otsuka et al., 2011). Demars et al. (2013) identified two new mutations in the *BMP15* gene associated with a rise in litter size (LS) and ovulation rate (OR). Interestingly, homozygous *FecX* ${}^{Gr}$ /*FecX* ${}^{Gr}$ Grivette sheep and homozygous *FecX* ${}^{O}$ /*FecX* ${}^{O}$ Olkuska sheep exhibited hyperproliferation, which is the opposite of the sterility expressed by all other known *BMP15* homozygous mutations (Demars et al., 2013).

Eight point mutations of G1, G2, G3, G4, G5, G6, G7, and G8 have been detected in the *GDF9* gene of Belclare and Cambridge sheep breeds (Hanrahan et al., 2004), and only five mutations will lead to deduced amino acid exchanges: G1 – R87H, G4 – E241K, G6 – V332I, G7– V371M, and G8 – S395F (Polley et al., 2010). The first mutation (G1) occurs in exon 1, and others (G4, G6, G7, and G8) occur in exon 2 of the *GDF9* gene. As for the *BMP15* gene there were identified four polymorphisms across the coding region (B1, B2, B3, and B4). But only two of them led to changes in the amino acid sequence, potentially affecting the fertility and sterility of sheep. Hanrahan et al. (2004) showed that only the G8 mutation in *GDF9* and the B2 and B4 mutations in *BMP15* were associated with the sterility phenotype. All sheep homozygous for G8 were sterile, all sheep homozygous for B2 or homozygous for B4 were sterile, and sheep heterozygous for both B2 and B4 simultaneously (i.e., one allele with each mutation, B2/B4) were sterile (Hanrahan et al., 2004).

In this paper, we investigate the polymorphisms of the *GDF9* and *BMP15* genes in the Kazakh meat–wool sheep breed and identify their relationship with the reproductive function and productivity of animals. To the best of our knowledge, studies on mutations in the *GDF9* and *BMP15* genes have not been determined in Kazakh native sheep breeds. This is especially crucial for the Kazakh meat–wool sheep breed, considering it is multifetal. Thus, the purpose of this work was to assess the genetic polymorphism of the Kazakh meat–wool sheep breed based on the *GDF9* and *BMP15* candidate genes responsible for fertility, as well as to determine the frequency of various alleles and genotypes for these genes in the studied populations.

## 2 Materials and methods

### 2.1 Sample collection

The objects of the study were 2- to 3-year-old sheep of Kazakh meat–wool breed bred in the Kuatzhan ( n=75 females) and Dukeev ( n=225 (of them 155 females and 70 males)) collective farms in the Zhambyl district of the Almaty region of the Republic of Kazakhstan. The sheep's peripheral blood was used as a biological material. Blood samples were collected by a veterinarian in EDTA vacuum tubes. All animal care and experiments were approved by the Local Ethics Committee of the Institute of Genetics and Physiology SC MSHE RK (19 October 2021, Almaty, Kazakhstan). Further, the blood samples were delivered to the Laboratory of Animal Genetics and Cytogenetics of the RSE Institute of Genetics and Physiology in containers with refrigerant and stored in a freezer (at -25 ${}^{\circ }$ C) until they were used for DNA extraction.

### 2.2 DNA extraction and quality assessment

DNA isolation was performed using the DNA-sorb-B kit for DNA extraction from clinical materials (AmpliSens, Moscow, Russia) according to the manufacturer's protocol. The determination of quantitative indicators was carried out on the NanoDrop One (ThermoScientific, USA), while the concentration of isolated DNA was 60–100 ng µ L ${}^{-1}$ on average. DNA qualitative characteristics were checked through agarose gel electrophoresis. Purified DNA was stored in a freezer at -25 ${}^{\circ }$ C for a week before being used for PCR-RFLP (polymerase chain reaction–restriction fragment length polymorphism) analysis.

### 2.3 PCR-RFLP analysis

PCR-RFLP analysis was performed to check for G1 and G8 mutations for the *GDF9* gene and B2 and B4 mutations for the *BMP15* gene among the two studied populations of Kazakh meat–wool sheep breed. The PCR-RFLP analysis conditions for the four mutations are shown in Table 1.

**Table 1:** Primer sequences and PCR-RFLP analysis conditions for *GDF9* and *BMP15* genotyping.

No.	SNP	Fragment	Primer sequences	Annealing	Genotyping
		length		temperature	
1	*GDF9* gene (G1 mutation)	462 bp	F: GAAGACTGTATGGGGAAATG R: CCAATCTGCTCCTACACACCT	63 ${}^{\circ }$ C	*Hha1*
2	*GDF9* gene (G8 mutation)	139 bp	F: CTTTAGTCAGCTGAAGTGGGACAAC R: ATGGATGATGTTCTGCACCATGGTGTGAACCTGA	62 ${}^{\circ }$ C	*Dde1*
3	*BMP15* gene (B2 mutation)	141 bp	F: CACTGTCTTCTTGTTACTGTATTTCAATGAGAC R: GATGCAATACTGCCTGCTTG	63 ${}^{\circ }$ C	*Hinf1*
4	*BMP15* gene (B4 mutation)	153 bp	F: GCCTTCCTGTGTCCCTTATAAGTATGTTCCCCTTA R: TTCTTGGGAAACCTGAGCTAGC	64 ${}^{\circ }$ C	*Dde1*

The digested fragments were checked using a 4 % agarose gel and visualized with ethidium bromide staining. The gels were scored for the presence or absence of the mutations by iBright Imaging Systems (ThermoScientific, USA). The statistical analysis of the obtained data was conducted using POPGENE 1.32 (https://mybiosoftware.com/popgene-1-32-population-genetic-analysis-2.html, last access: 15 November 2023).

### 2.4 Sequencing of *GDF9* and *BMP15* genes and data analysis

Sequencing of exon regions of the *GDF9* and *BMP15* genes was carried out to build a genetic tree in comparison with sequence data of these genes from other sheep populations and to confirm the existence or absence of the desired mutations in the studied populations. For this purpose, 15 samples selected according to agarose gel visuals were subjected to sequence analysis. First, the *GDF9* and *BMP15* sheep genes were amplified using PCR with primers designed on the IDT website (https://eu.idtdna.com/pages/tools/primerquest, Integrated DNA technologies, 2023) from published sheep sequences (sheep genomic *BMP15* exon 1, AF236078; sheep genomic *BMP15* exon 2, AF236079; sheep genomic *GDF9* exons 1 and 2, AF078545). The resulting PCR products were sequenced on an ABI 3500XL sequencer (Applied Biosystems) using BigDye™ Terminator v3.1 Cycle Sequencing Kit and the BigDye XTerminator™ Purification Kit. Detailed information on the primers is shown in Table 2.

**Table 2:** Primers used for the amplification of the *GDF9* and *BMP15* genes.

No.	Primer name	Primer sequence	Sequencing	Annealing
			fragment	temperature
			length	
1	*GDF9* exon1	F: AGACCAACCGAGGCTCTT R: CCCATTAACCAATCTGCTCCTAC	503 bp	57 ${}^{\circ }$ C
2	*GDF9* exon2 primer1	F: TCTGAGGTACTGATTCCTTGATTT R: CCCTCAGCAGCTTCTTCTC	604 bp	56 ${}^{\circ }$ C
3	*GDF9* exon2 primer2	F: TGAACGACACAAGTGCTCAG R: GACACATGAAACTTCCTCCCA	649 bp	57 ${}^{\circ }$ C
4	*BMP15* exon1	F: AGAACATGTTGCTGAACACC R: TGAGAGGCCTTGCTACAC	354 bp	55 ${}^{\circ }$ C
5	*BMP15* exon2 primer1	F: CACATACAGACCCTGGACTTTC R: CTTTAGGGAGAGGTTTGGTCTTC	418 bp	57 ${}^{\circ }$ C
6	*BMP15* exon2 primer2	F: GAAGACCAAACCTCTCCCTAAAG R: CTGGGCAATCATACCCTCATAC	436 bp	57 ${}^{\circ }$ C

Obtained sequence results belonging to the mutant and wild-type alleles were aligned and compared with the *GDF9* sequences of *Ovis aries* from Sudan, USA, Brazil, Türkiye, Mexico, Norway, China, Iraq, and Egypt (GenBank accession numbers listed in the phylogenetic tree), using BioEdit 7.7 (Alzohairy, 2011). The phylogenetic tree was constructed using the neighbor-joining method on MEGA software (https://www.megasoftware.net/, Molecular Evolutionary Genetics Analisys, 2023).

## 3 Results and discussion

### 3.1 PCR-RFLP analysis

The G1 mutation causes an increase in ovulation rate or multiple births in heterozygous ewes. However, for homozygous ewes, it causes sterility because of the arrested follicular development (Hanrahan et al., 2004). Some authors reported that heterozygous ewes with the G1 mutation were fertile and had a large litter size, while wild-type ewes had a smaller litter size (Abdoli et al., 2013). Another study revealed that ewes of the AG genotype have a higher number of lambs (1.88) compared to AA genotypes (1.22) (Gorlov et al., 2018). Paz et al. (2015) reported that the G1 mutation increased the ovulation rate in heterozygous ewes in Chilota sheep. Apart from its effects on ovulation rates, it was reported that lamb weight at birth in heterozygous ewes was higher than in homozygous ewes (Getmantseva et al., 2019). Liandris et al. (2012) revealed that the G1 and G8 mutations of the *GDF9* gene were only significantly over-presented in the highly prolific Greek sheep breed Chios.

Regarding the sterility of sheep, Hanrahan et al. (2004) revealed that only the G8 change in *GDF9* and the B2 and B4 changes in *BMP15* were associated with the sterility phenotype. Thus, homozygous individuals of sheep that do not have any of the three mutations sought are fertile ( + / + ), sheep heterozygous for one of the three mutations have an increased ovulation rate (G8/ + ; B2/ + or B4/ + ), and homozygous carriers of one of the three mutations of the individual are sterile (G8/G8; B2/B2 or B4/B4). However, it is also crucial to consider the relationship of these mutations to each other: individuals that are heterozygous at the same time for mutations B2 (B2/ + ) and B4 (B4/ + ) are sterile, and heterozygous sheep for G8 (G8/ + ) and B2 (B2/ + ) or G8 (G8/ + ) and B4 (B4/ + ) simultaneously have an even higher ovulation rate. Also, studies report that the effect of the *GDF9* mutation is significantly higher than that of the *BMP15* mutations. However, the *GDF9* and *BMP15* mutations have an additive effect while appearing in one animal simultaneously, implying that *GDF9* and *BMP15* are likely to work independently (Hanrahan et al., 2004).

**Figure 1:**
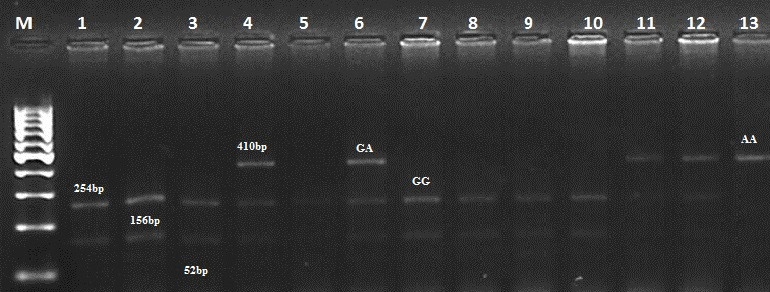
Determination of genotypes for the *GDF9* gene (mutation G1). M – GeneRuler DNA Ladders (ThermoScientific, USA). 1–13 – digested fragments.

Considering this information, we selected four mutations (G1, G8, B2, and B4) for PCR-RFLP analysis. We analyzed 300 individuals of the Kazakh meat–wool sheep breed from two distinct populations for these mutations. As for the G1 mutation, the nucleotide substitution G to A in the *GDF9* exon1 region disrupts the cleavage site of the *HhaI* restriction enzyme (GCGC to GCAC) at nucleotide 260 of the 462 bp PCR product. Thus, after the treatment of a 462 bp PCR product with the Hha1 enzyme, three fragments of 254, 156, and 52 bp were observed for the wild homozygous GG genotype; four fragments of 410, 254, 156, and 52 bp for the mutant heterozygous GA genotype; and two fragments of 410 and 52 bp for the mutant homozygous AA genotype (Fig. 1).

As for the G8 mutation, the 139 bp PCR fragment was observed with two *DdeI* restriction sites in the AA genotype at 31 and 108 bp (wild type). The results indicated no polymorphism in this exon among the studied sheep of the Kazakh meat–wool breed. The G8 mutation was also not found in other populations of sheep considered to be prolific: Kermani (Khodabakhshzadeh et al., 2016) and Shal sheep in Iran (Ghaffari et al., 2009), Merino sheep in Germany (Chu et al., 2005) and China (Guan et al., 2005), and others (Bai et al., 2007; Sun et al., 2009). Thus, it can be assumed that the G8 mutation is not associated with litter size in Kazakh meat–wool sheep, as in the abovementioned sheep breeds.

**Table 3:** Distribution of genotypes for the *GDF9* gene (mutation G1) in two populations of Kazakh meat–wool sheep breed.

Population	Overall	Identified genotypes			Allele frequency		Genotype frequency		
		GG	GA	AA	G	A	GG	GA	AA
Kuatzhan collective farm	75	60	14	1	0.89	0.11	0.79	0.196	0.012
Dukeev collective farm	225	176	45	4	0.88	0.12	0.78	0.21	0.01
Overall	300	236	59	5	0.88	0.12	0.78	0.21	0.01
		$\chi^{2}=2.553$							

The following data were used to detect the mutations in the *BMP15* gene: processing of a 141 bp fragment of the *BMP15* gene with the *Hinf1* restriction enzyme results in two fragments of 106 and 35 bp (in the presence of mutation B2), and processing of a 153 bp fragment with *Dde1* restriction enzyme forms two fragments of 118 and 35 bp (in the presence of mutation B4). Thus, in the mutation's presence, three fragments should be detected on the electrophoregram: a fragment with the original length of the PCR product and two fragments subjected to restriction enzyme processing. However, the third 25–35 bp fragment cannot be seen visually. The wild type, which does not have the desired mutation, is not subjected to restriction enzyme treatment. Therefore, only one fragment is detected on the electrophoregram, which kept the original size of the PCR product. The study results showed that B2 and B4 single-nucleotide polymorphisms (SNPs) of the *BMP15* gene were monomorphic in the Kazakh meat–wool sheep breed populations, which is consistent with the results of the Iran-Black sheep population (Rezaei et al., 2020). Liandris et al. (2012) reported that the B4 mutation of the *BMP15* gene was only significantly over-presented in the less prolific Greek sheep breed Karagouniki. Thus, we may suggest that this mutation does not determine the level of fertility in some sheep breeds, including the Kazakh meat–wool breed. This assumption should be tested in further studies aimed at studying the effect of this mutation on the fertility of the Kazakh meat–wool sheep breed by comparing the genotypic data with the phenotypic ones. It is also crucial to understand that the presence or absence of these mutations at the same time is not an indicator of increased fertility or sterility in animals, as in the study of Iranian fat-tailed sheep, where prolificacy genes were polymorphic in the studied population (Abdoli et al., 2013).

Regarding the G1 mutation of the *GDF9* gene, 78.7 % of the studied sheep were homozygous for the wild type (GG genotype). Thus, the studied mutation occurred only in 1.6 % of sheep in the homozygous form (G1/G1, genotype AA) and 19.7 % of animals in the heterozygous state (G1/ + , genotype GA). There were no differences in these parameters among the two populations (Table 3).

These results are consistent with Kirikçi et al. (2021), where 21 % of the studied Turkish Karayaka sheep were heterozygous GA genotype carriers. However, in our study, the mutant homozygous AA genotype was also found, albeit in a small amount. The homozygous mutant genotype AA was also found among Sudanese desert sheep breeds, and its frequency was equal to 0.06. It was reported that the ewes with heterozygous (GA) and homozygous wild-type (GG) genotypes had more lambs than the homozygous (AA) genotypes (Abdelgadir et al., 2021) compared with the Kazakh meat–wool breed, where this indicator was equal to 0.01. A few number of homozygous individuals for the mutant genotype can be explained by the low frequency (0.12) of the A allele in the Kazakh meat–wool sheep breed, and mutant genotypes could suffer from embryonic death and reproductive defects. The obtained result might also be due to differences in the biological effects of mutations by species (Dinçel et al., 2018; Javanmard et al., 2011).

The data obtained were analyzed for the determination of several statistical indicators: the identified and effective number of alleles, Nei's gene diversity, Shannon's information index, and original measures of Nei's genetic identity and genetic distance (Table 4).

**Table 4:** Summary of the genetic variation statistics for all loci of the G1 mutation of the *GDF9* gene.

No.	Population	Overall	Locus	$N_{a}$	$N_{e}$	h	I
1	Kuatzhan collective farm	75	GG	2.0000	1.8276	0.4528	0.6452
			GA	2.0000	1.0850	0.0783	0.1706
			AA	2.0000	1.0412	0.0396	0.0988
			Mean	2.0000	1.3179	0.1902	0.3049
			SD	0.0000	0.4419	0.2282	0.2969
2	Dukeev collective farm	225	GG	2.0000	1.9998	0.4999	0.6931
			GA	2.0000	1.2817	0.2198	0.3781
			AA	2.0000	1.0089	0.0089	0.0286
			Mean	2.0000	1.4301	0.2429	0.3666
			SD	0.0000	0.5118	0.2463	0.3324

$N_{a}$ : observed number of alleles. $N_{e}$ : effective number of alleles. h : Nei's gene diversity. I : Shannon's information index.

The average value of the observed number of alleles ( $N_{a}$ ), representing the actual number of alleles found in the studied populations, was the same (2.0000) for all identified genotypes in both populations. The average effective number of alleles ( $N_{e}$ ), describing the number of alleles with the same frequency required to achieve the same expected heterozygosity as in the population under study, varied from 1.0089 to 1.9998, while in the second population it was higher, possibly because of the difference in the studied animals' number among the populations. In terms of gene diversity ( h ), it can be noted that a significant difference in this indicator was observed for the AA locus, 0.0396 and 0.0089, in the first and second populations, respectively. Shannon's index ( I ), which measures gene diversity, showed that the genetic diversity of the Dukeev collective farm population was higher than that of the Kuatzhan collective farm population, at 0.3324 and 0.2969, respectively. Both populations were found to be in Hardy–Weinberg disequilibrium ( $\chi^{2}=2.553$ ) because of the heterozygosity deficit. Possible causes of heterozygosity deficits are inbreeding, selection, sub-population structure, and genetic drift.

**Figure 2:**
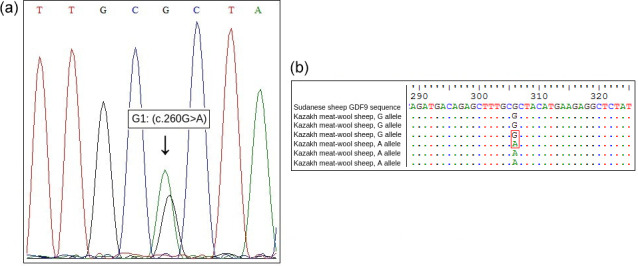
**(a, b)** Identified SNP in the exon 1 of *GDF9* gene in Kazakh meat–wool sheep breed.

### 3.2 Sequencing of *GDF9* and *BMP15* genes

Sequencing of exon regions of *GDF9* in 15 Kazakh meat–wool sheep was performed to confirm the G1 mutation of the *GDF9* gene detected by PCR-RFLP analysis. Sequencing results for both alleles were compared to the *GDF9* sequencing results for Sudanese sheep (GenBank accession: KY310682.1) to show the nucleotide change (Fig. 2).

Abdelgadir et al. (2021) revealed that the presence of one copy of the G1 mutation of *GDF9* gene increased litter size in the studied Sudanese Desert sheep, and this locus may be used as a biomarker for litter size improvement through genotypic selection and allele or gene introgression.

**Figure 3:**
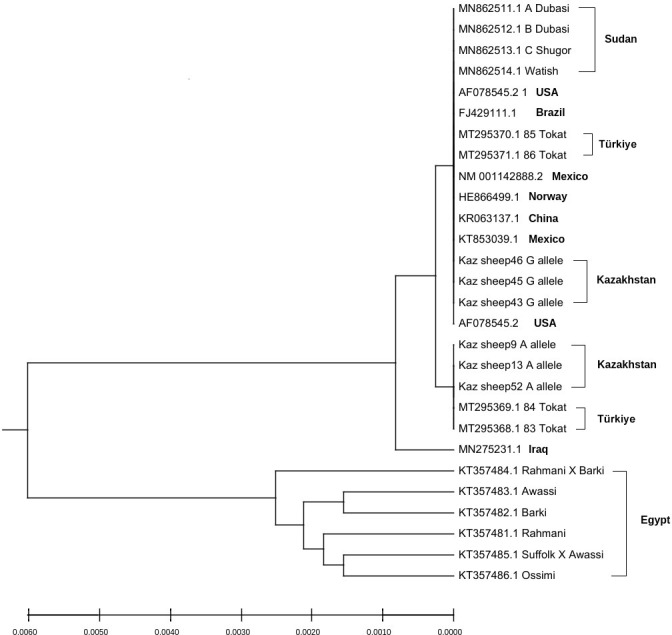
Phylogenetic tree based on *GDF9* gene of different sheep breeds.

Sequences of exon 1 of the *GDF9* gene of different sheep breeds with high identities were downloaded from NCBI in the FASTA format and used for phylogenetic tree construction. Thus, wild individuals (genotype GG) and carriers of the G1 mutation (genotype AG) settled down on separate branches of the phylogenetic tree (Fig. 3).

Remarkably, the carriers of the G allele (wild type) were in the same branch of the phylogenetic tree with Sudanese Dubasi, Shugor, and Watish sheep breeds, which had the lowest frequencies of G1 mutant genotypes (Abdelgadir et al., 2021). Also, there were other sheep breeds in which the G1 mutation was not identified, such as Brazilian Santa Inês sheep (Silva et al., 2011) and Norwegian white sheep (Våge et al., 2013) (Muñoz-García et al., 2021; Bodensteiner et al., 1999), whereas the carriers of the A allele (mutant type) are on a close branch with the carriers of this mutation in Turkish sheep breeds (Kirikçi et al., 2021). Egyptian sheep breeds, in which the G1 mutation was also not found (Saleh, 2020), are on a branch of the phylogenetic tree separate from all other sheep breeds. The sequences of the exon2 of the *GDF9* gene and the *BMP15* (exon1 and exon2) were also used for phylogenetic tree construction to determine the relatedness of the Kazakh meat–wool breed with other world sheep populations according to the studied genes (Figs. S1 and S2 in the Supplement). Thus, it was revealed that the Kazakh meat–wool sheep breed has a close phylogenetic relationship with sheep of Mexican origin in the *GDF9* gene (exon 2) and with sheep of Iranian and Brazilian origin in the *BMP15* gene.

The present results have important practical implications for the Kazakh meat–wool sheep breed. The application of a marker-assisted selection scheme could be pursued, with introgression of the favored allele(s) showing positive dominance effects on litter size. It could lead to a significant improvement in fecundity, with apparent economic implications for the breeders.

## 4 Conclusion

Considering the findings from the present study, fertility traits might be improved in the Kazakh meat–wool sheep breed, as evidenced in other sheep breeds. However, it is necessary to perform further studies to provide exact evidence of the effects of the G1 mutation on fertility traits in the Kazakh meat–wool sheep breed. In conclusion, we determined that exon 1 of the *GDF9* gene was polymorphic in the Kazakh meat–wool sheep breed and found the G1 mutation (c.260G > A) on this gene. This mutation was reported to have the relationships with the animals' litter size in other sheep breeds. For this reason, similar relationships should be investigated in Kazakh meat–wool sheep.

## Supplement

10.5194/aab-66-401-2023-supplement The supplement related to this article is available online at: https://doi.org/10.5194/aab-66-401-2023-supplement.

## Data Availability

and gene sequencing data of Kazakh meat–wool sheep used in this study are available from GenBank accession no. PRJNA1001622 ((https://www.ncbi.nlm.nih.gov/bioproject/PRJNA1001622/, Institute of Genetics and Physiology, 2023).
