# Supplementary material for: Detection of genetic variations in the GDF9 and BMP15 genes in Kazakh meat–wool sheep
Source: Arch Anim Breed. 2023 Dec 7;66(4):401–9. doi: 10.5194/aab-66-401-2023 (PMC10776884; doi:10.5194/aab-66-401-2023)
Supplement: The supplement related to this article is available online at: https://doi.org/10.5194/aab-66-401-2023-supplement. [file aab-66-401-supplement.pdf]

Supplement of Arch. Anim. Breed., 66, 401–409, 2023
https://doi.org/10.5194/aab-66-401-2023-supplement
© Author(s) 2023. CC BY 4.0 License.

Open Access
Archives Animal Breeding

*Supplement of*

# Detection of genetic variations in the *GDF9* and *BMP15* genes in Kazakh meat–wool sheep

**Makpal Amandykova et al.**

*Correspondence to:* Makpal Amandykova (makpal_30.01@mail.ru) and Kairat Dossybayev (kairat1987_11@mail.ru)

The copyright of individual parts of the supplement might differ from the article licence.

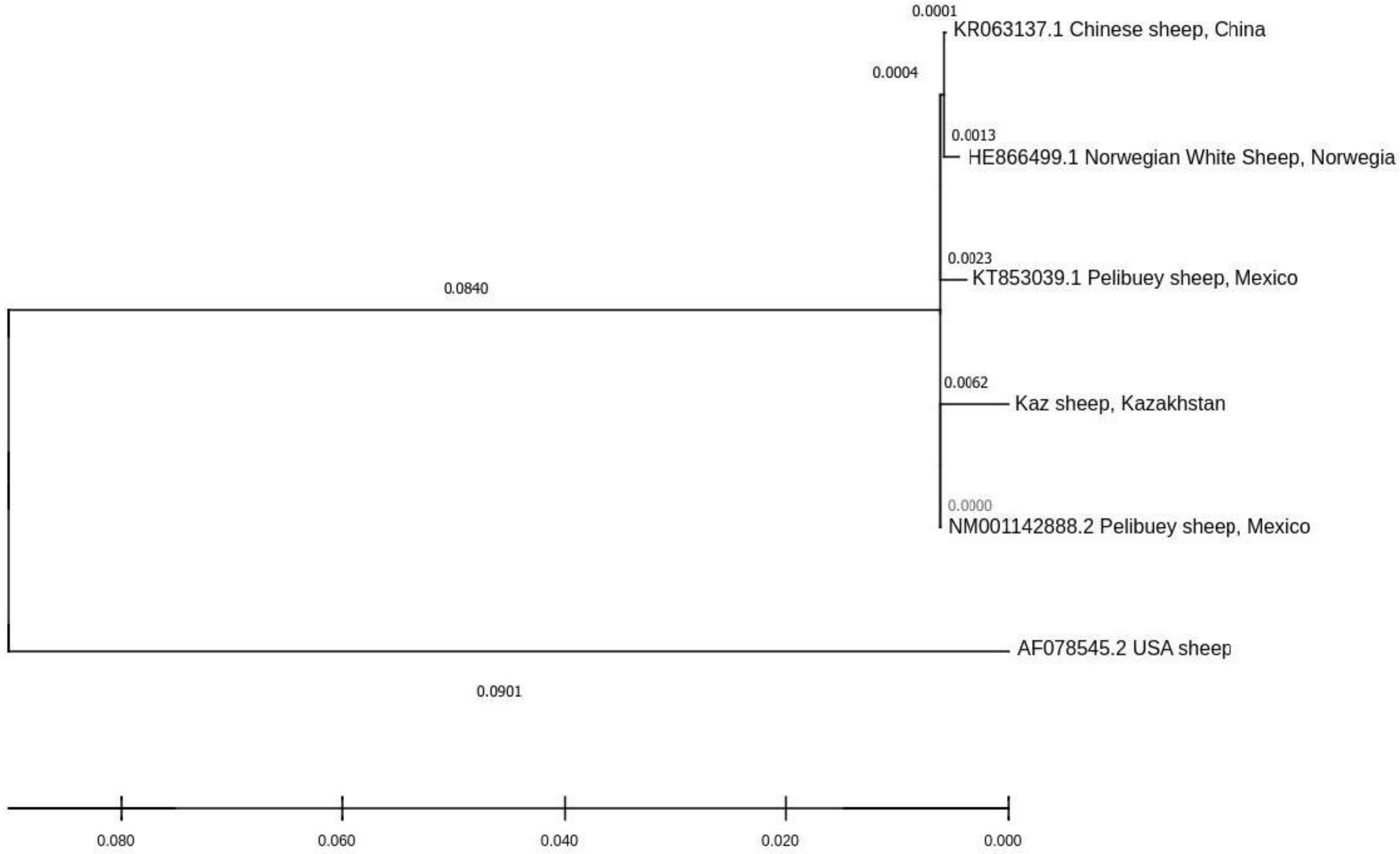


**S1. Phylogenetic tree based on GDF9 gene (Exon 2) of different sheep breeds.**

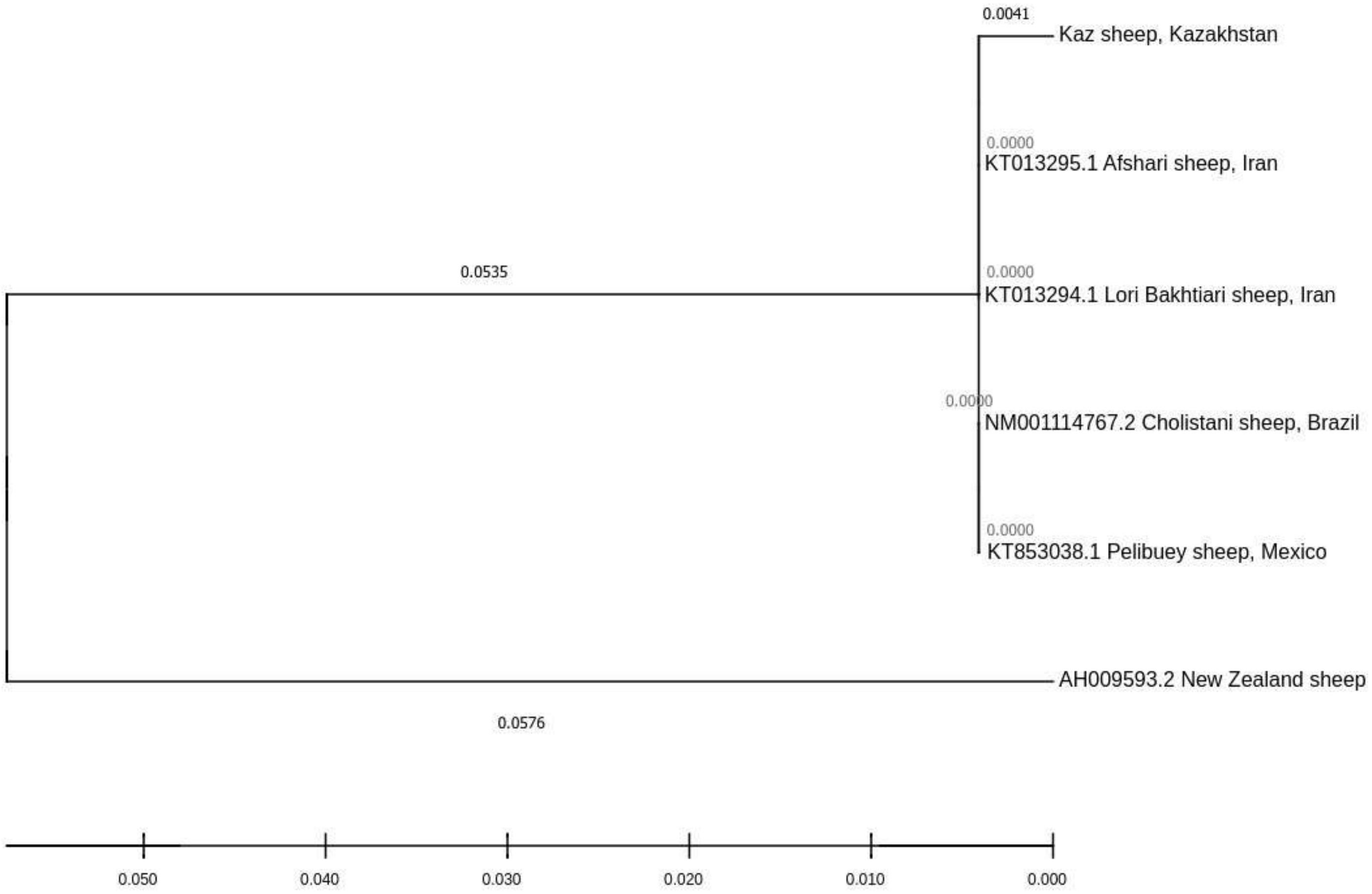


**S2. Phylogenetic tree based on BMP15 gene of different sheep breeds.**
